# Upregulation of CCT3 predicts poor prognosis and promotes cell proliferation via inhibition of ferroptosis and activation of AKT signaling in lung adenocarcinoma

**DOI:** 10.1186/s12860-022-00424-7

**Published:** 2022-06-30

**Authors:** Kun Wang, Jian He, Changling Tu, Hui Xu, Xugang Zhang, Yongchang Lv, Chao Song

**Affiliations:** 1 Yunnan Kungang Hospital, Kunming Fourth People’s Hospital, Seventh Affiliated Hospital of Dali University, No 9. Ganghe south Road, Anning City, Kunming, 650301 China; 2 The Third Affiliated Hospital of Kunming Medical University, Kunming, China

**Keywords:** LUAD, CCT3, slc7a11, Ferroptosis, AKT

## Abstract

**Background:**

Chaperonin containing TCP1 subunit 3 (CCT3) acts as an oncogene in cancers, whereas its role and underlying mechanisms in lung adenocarcinoma (LUAD) are poorly understood. This study investigated the clinical relevance and function of CCT3 in LUAD.

**Methods:**

Clinical relevance of CCT3 in LUAD and lung squamous cell carcinoma (LUSC) was analyzed based on TCGA database. qRT-PCR and Western blot were used to detect mRNA and protein expression, respectively. CCK8 and colony formation were performed to measure cell viability. PI and PI/Annexin V-FITC assay kit was used to determine cell cycle and cell death, respectively. Luciferase activity was performed to check whether CCT3 regulated slc7a11’s transcription activity. Ferroptosis was determined by incubating the cells with ferroptosis and apoptosis inducer, their inhibitor and autophagy inhibitor, followed by cell viability examination.

**Results:**

We found that CCT3 was overexpressed in LUAD and LUSC tissues. Overexpression of CCT3 predicted the poor prognosis of LUAD patients. Loss-of-function and gain-of-function experiments demonstrated that CCT3 promoted the proliferation and colony formation of LUAD cells. In addition, CCT3 promoted cell cycle progression and suppressed slc7a11-mediated cell ferroptosis, but not apoptosis. We also found that CCT3 activated AKT. MK2206 significantly reduced the viability of CCT3 overexpressed LUAD cells, while had smaller inhibitory effect on the proliferation of control cells, suggesting that CCT3 dictates the sensitivity of LUAD cells to AKT inhibition.

**Conclusion:**

Our study demonstrates that CCT3 contributes to the proliferation and growth of LUAD cells through inhibition of ferroptosis and activation of AKT.

**Supplementary Information:**

The online version contains supplementary material available at 10.1186/s12860-022-00424-7.

## Introduction

Lung cancer is the most lethal malignancy, causing approximately 160 000 death per year globally [1]. More than 80% of lung cancer patients are non-small cell lung cancer (NSCLC) and lung adenocarcinoma (LUAD) represents the major histologic subtype of NSCLC [2]. Besides, NSCLC also includes lung squamous cell carcinoma (LUSC) and large cell carcinoma [3]. The five-year overall survival of NSCLC is very poor. For the past decades, genetic, transcriptional and proteomics studies have been performed to illustrate the molecular and pathological events responsible for NSCLC development. As a result, targeted therapy, immunotherapy, and the combination therapy have become the promising treatment strategies for this malignancy [4–6]. However, only a small percentage of the patients benefit from the therapies and most of them relapse after years. Therefore, understanding the molecular mechanisms triggering this malignancy can help us develop effective drug target to cure this deadly disease.

Chaperonin containing TCP1 subunit 3 (CCT3) belongs to TCP1 ring complex (TRiC) family, which consists of eight subunits, including CCT1-CCT8 [7, 8]. It has been shown that TRiC family regulates the folding of cytoskeletal proteins, such as tubulin and actin [9, 10]. Upregulation of TRiC has an important effect on cell cycle progression by regulating plk1 [11]. As a subunit of TRiC family, CCT3 is a promising diagnostic biomarker for cancers and dysregulation of CCT3 contributes to cancer progression. CCT3 is highly expressed in hepatocellular carcinoma (HCC) tissues and promotes the tumorigenesis of HCC [12, 13]. CCT3 is also overexpressed in gastric cancers. Knockdown of CCT3 reduces the viability of gastric cancer cells by regulating cell cycle proteins [14]. Furthermore, CCT3 overexpression contributes to the development of NSCLC through regulating YAP1 and promotes the resistance of cisplatin in LUAD cells through JAK2/STAT3 signaling pathway [15, 16]. These results suggest that CCT3 plays an important role in cancer development. Even though two studies have illustrated the function of CCT3 in lung cancer, the precise role and molecular mechanism of CCT3 in LUAD needs more experiments to be determined. Understanding how CCT3 contributes to LUAD progression may help us develop effective drugs to treat this deadly malignancy.

Herein, we explored the prognostic value of CCT3 by analyzing the expression of CCT3 in LUAD and LUSC tissues and its correlation with the prognosis of lung cancer patients. We also performed loss-of-function and gain-of-function experiments to study the role of CCT3 in LUAD cell growth and proliferation. Our study suggests that CCT3 acts as an oncogene in LUAD.

## Results

### CCT3 is upregulated in lung cancer tissues

**Fig. 1:**
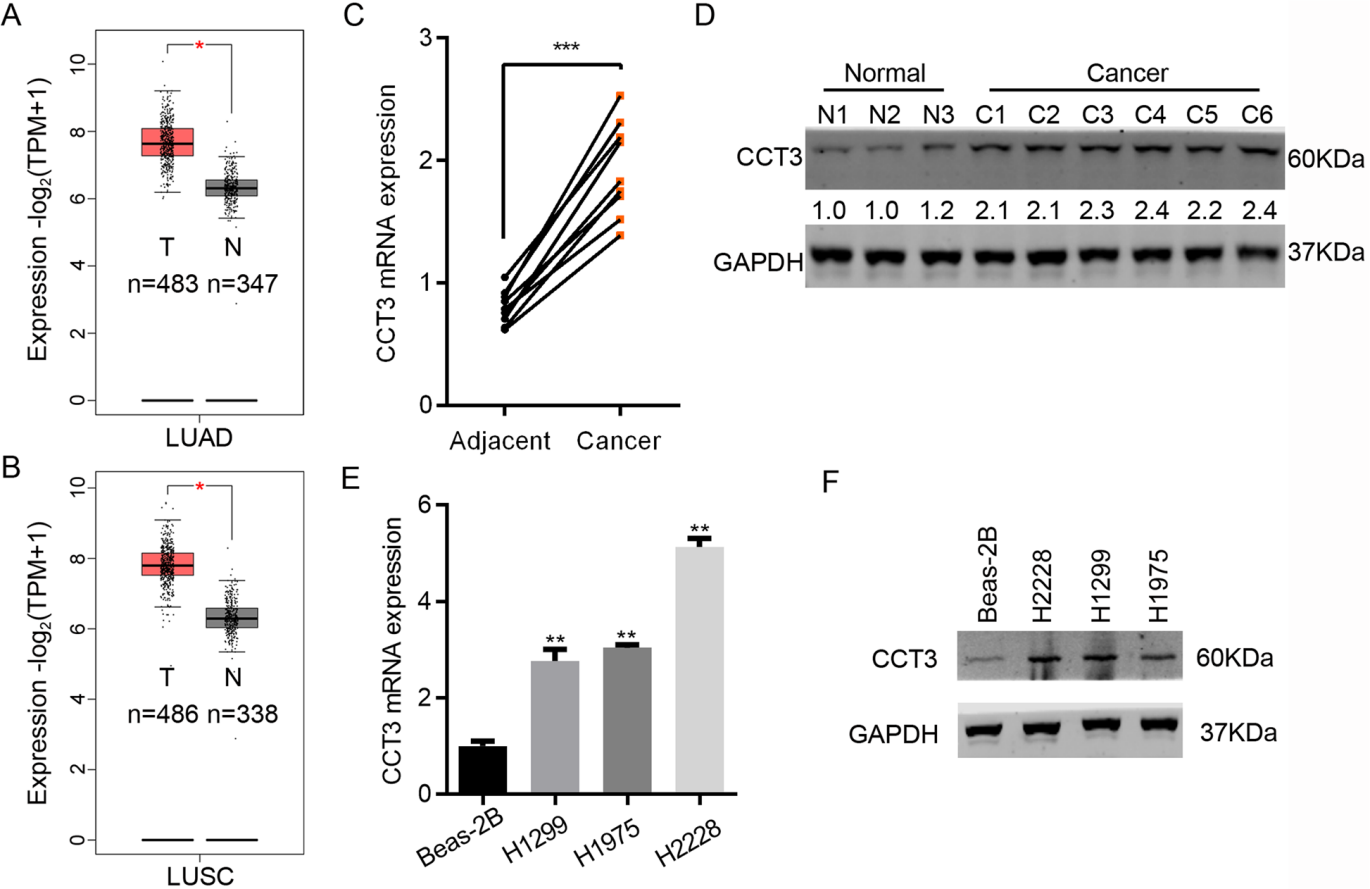
CCT3 is overexpressed in lung cancer tissues and cells. **A** The transcript abundance of CCT3 was analyzed in lung adenocarcinoma (LUAD, *n* = 483) and normal tissues (*n* = 347) from TCGA database. *p < 0.05. **B** The transcript abundance of CCT3 was analyzed in lung squamous cell carcinoma (LUSC, *n* = 486) and normal tissues (*n* = 338) from TCGA database. *p < 0.05. **C** Nine pairs of lung cancer and adjacent normal tissues were subjected to qRT-PCR analysis of CCT3 expression. ***p < 0.001. **D** Western blot analysis of CCT3 in lung cancer and paired adjacent normal tissues. The blots were cut prior to hybridisation with antibodies during blotting. **E** and **F** qRT-PCR (**E**) and Western blot (**F**) analysis of CCT3 in normal lung cells Beas-2B and in cancer cells H2228, H1299 and H1975. **p < 0.01. GAPDH is the internal control. The blots were cut prior to hybridisation with antibodies during blotting

To explore the role of CCT3 in lung cancer, we first analyzed CCT3 based on TCGA database. We found that CCT3 was significantly upregulated in lung adenocarcinoma (LUAD) and lung squamous cell carcinoma (LUSC) tissues (Fig. 1A and B). Then lung cancer and paired adjacent normal tissues were collected for the examination of CCT3 mRNA and protein abundance. The results showed that the transcript of CCT3 was increased in cancer tissues compared with normal tissues (Fig. 1C). Western blot results showed that CCT3 was upregulated at protein level in cancer tissues (Fig. 1D). Furthermore, we detected the mRNA and protein expression of CCT in normal lung cells and cancer cells. The results showed that CCT3 was highly expressed in lung cancer cells, including H2228, H1299 and H1975, as compared with normal cells Beas-2B (Fig. 1E and F).

### CCT3 expression predicts poor prognosis of LUAD but not LUSC patients

**Fig. 2:**
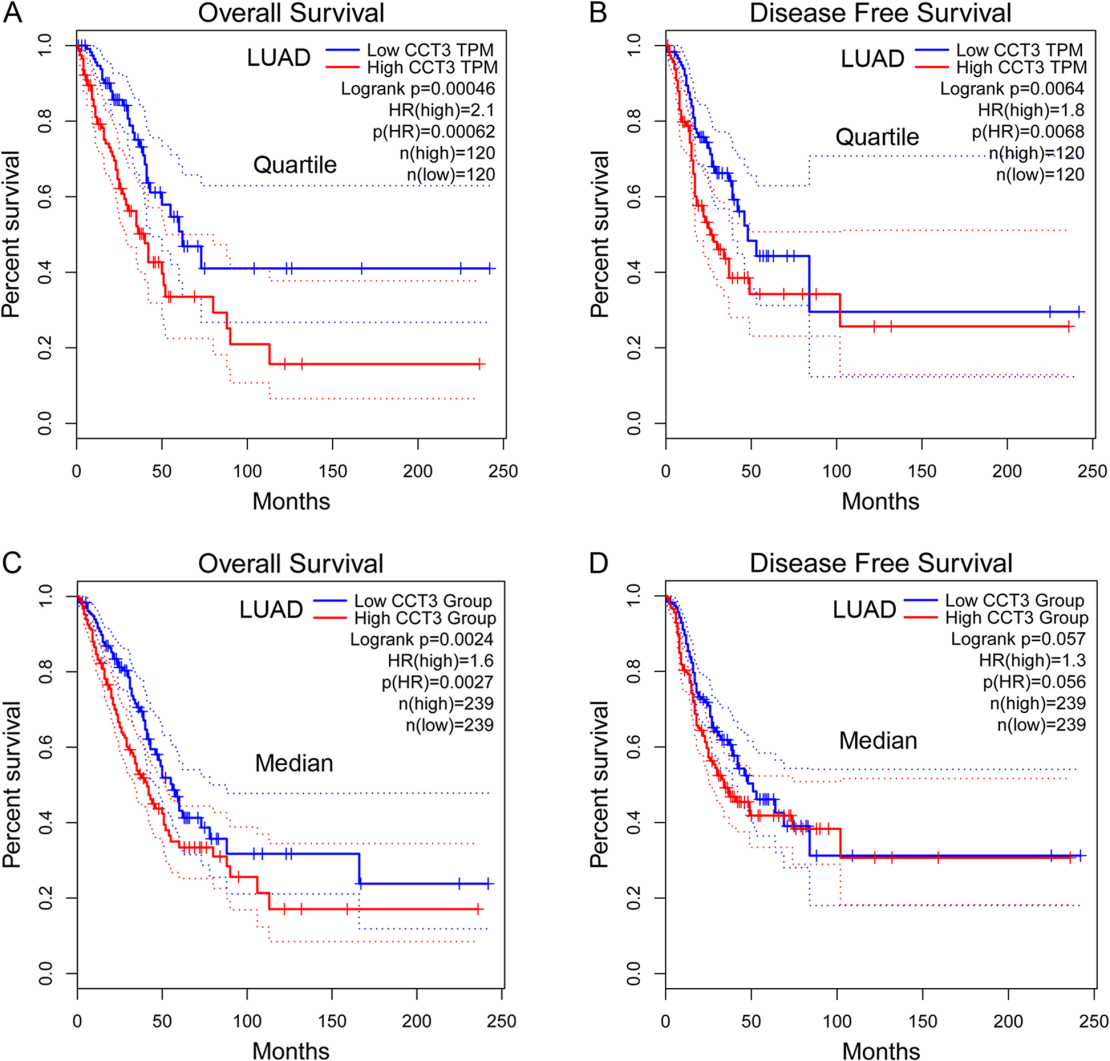
CCT3 expression is associated with the survival of LUAD patients. **A** Overall survival of LUAD patients with high expression and low expression of CCT3. *n* = 120 in each group. *p* = 0.00046. **B** Disease free survival of LUAD patients with high expression and low expression of CCT3. *n* = 120 in each group. *p* = 0.0064. The data analysis was performed using Quartile. **C** Overall survival of LUAD patients with high expression and low expression of CCT3. *n* = 239 in each group. *p* = 0.0027. **D** Disease free survival of LUAD patients with high expression and low expression of CCT3. *n* = 239 in each group. *p* = 0.057. The data analysis was performed using Median

Lung cancer is the most lethal malignancy worldwide. We next investigated the significance of CCT3 on the survival of LUAD and LUSC patients. Based on the data from TCGA, we found that CCT3 expression was correlated with the prognosis of LUAD patients. Firstly, the data analysis was performed using Quartile. LUAD patients with overexpressed CCT3 had shorter overall and disease-free survival than the patients with lowly expressed CCT3 (Fig. 2A, *p* = 0.00062 and Fig. 2B, *p* = 0.0068). Secondly, the data analysis was performed using Median. LUAD patients with overexpressed CCT3 had shorter overall survival than the patients with lowly expressed CCT3 (Fig. 2C, *p* = 0.0027). However, there was no significant difference when analyzing disease-free survival (Fig. 2D, *p* = 0.057). We further analyzed the correlation between CCT3 expression and LUSC patients’ prognosis. The results showed that there was no significant relationship between CCT3 expression and the overall and disease-free survival of LUSC patients (Supplementary Fig. 1). Taken together, CCT3 is associated with the progression of LUAD but not LUSC.

### CCT3 contributes to the growth and migration of LUAD cells

**Fig. 3:**
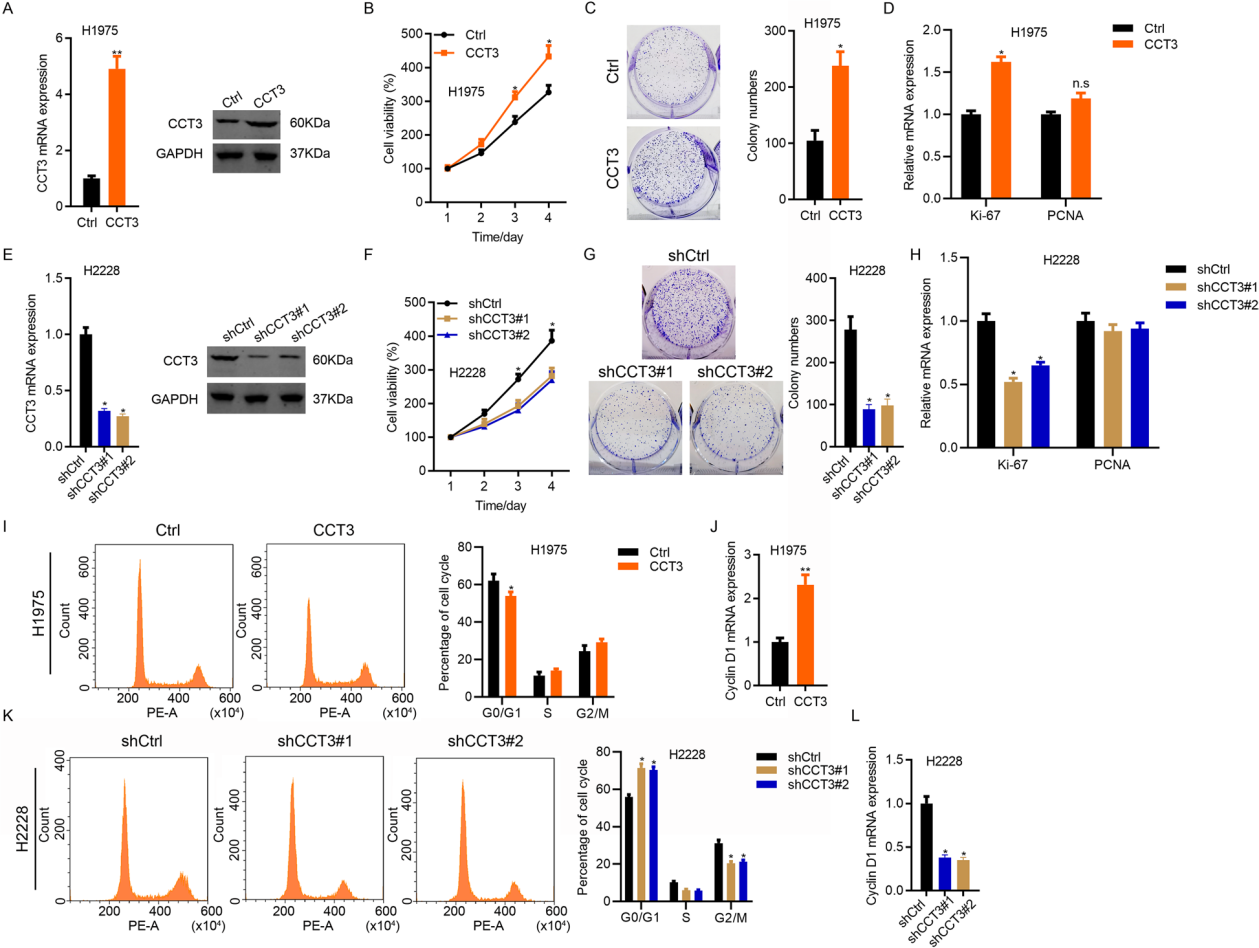
CCT3 promotes the growth and cell cycle progression of LUAD cells. **A** H1975 cells were infected with Ctrl and CCT3 overexpression lentivirus. qRT-PCR and Western blot analysis of CCT3 in Ctrl and CCT3 overexpressed H1975 cells. GAPDH is the internal control. **p < 0.01. The blots were cut prior to hybridisation with antibodies during blotting. **B** Ctrl and CCT3 overexpressed H1975 cells were subjected to CCK8 analysis of cell proliferation. *p < 0.05. **C** Colony formation analysis was performed on Ctrl and CCT3 overexpressed H1975 cells. *p < 0.05. **D** qRT-PCR analysis of Ki-67 and PCNA in Ctrl and CCT3 overexpressed H1975 cells. GAPDH is the internal control. *p < 0.05. **E** H2228 cells were infected shCtrl, shCCT3#1 and shCCT3#2 knockdown lentivirus. qRT-PCR and Western blot analysis of CCT3 in shCtrl, shCCT3#1 and shCCT3#2 H2228 cells. GAPDH is the internal control. *p < 0.05. The blots were cut prior to hybridisation with antibodies during blotting. **F** shCtrl, shCCT3#1 and shCCT3#2 H2228 cells were subjected to CCK8 analysis of cell proliferation. *p < 0.05. **G** Colony formation analysis was performed on shCtrl, shCCT3#1 and shCCT3#2 H2228 cells. *p < 0.05. **H** qRT-PCR analysis of Ki-67 and PCNA in shCtrl, shCCT3#1 and shCCT3#2 H2228 cells. GAPDH is the internal control. *p < 0.05. **I** Cell cycle was analyzed by PI staining in Ctrl and CCT3 overexpressed H1975 cells. Left, representative images of cell cycle. Right, quantification of cell cycle. *p < 0.05. **J** qRT-PCR analysis of cyclin D1 in Ctrl and CCT3 overexpressed H1975 cells. GAPDH is the internal control. **p < 0.01. **K** Cell cycle was analyzed by PI staining in shCtrl, shCCT3#1 and shCCT3#2 H2228 cells. Left, representative images of cell cycle. Right, quantification of cell cycle. *p < 0.05. **J** qRT-PCR analysis of cyclin D1 in shCtrl, shCCT3#1 and shCCT3#2 H2228 cells. GAPDH is the internal control. **p < 0.01

Although CCT3 was upregulated in both LUAD and LUSC tissues, CCT3 high expression was only related with the prognosis of LUAD patients. We then aimed to examine the effect of CCT3 on LUAD cell function through gain-of-function and loss-of-function experiments. As shown in Fig. 1F, CCT3 expression kept highest in H2228 cells and moderate in H1975 cells. Thus, we overexpressed CCT3 in H1975 cells and knocked down CCT3 in H2228 cells. qRT-PCR and Western blot results demonstrated that CCT3 was ectopically expressed in H1975 cells compared with the control cells (Fig. 3A). CCK8 experiments showed that CCT3 overexpression promoted the proliferation of H1975 cells (Fig. 3B). Consistently, CCT3 upregulation enhanced the colony growth ability of H1975 cells (Fig. 3C), suggesting that CCT3 contributes to LUAD cell proliferation. Next, we examined whether proliferation markers were regulated by CCT3. qRT-PCR results showed that the expression Ki-67, but not PCNA, was slightly upregulated in CCT3 overexpressed H1975 cells (Fig. 3D). To confirm the results, we downregulated CCT3 using two different shRNAs in LUAD cells H2228 and the knockdown efficiency was confirmed by qRT-PCR and immunoblotting results (Fig. 3E). We found that CCT3 silencing significantly suppressed the proliferation and colony formation of H2228 cells (Fig. 3F and G). In consistent with the qRT-PCR results in CCT3 overexpressed H1975 cells, CCT3 knockdown slightly downregulated Ki-67, while had no effect on the expression of PCNA (Fig. 3H).

Next, we investigated whether CCT3 modulated the cell cycle of LUAD cells by staining the cells with PI and subjected them to flow cytometry analysis. We found that CCT3 overexpression resulted in reduced G0/G1 phase in H1975 cells, while CCT3 knockdown led to increased G0/G1 phase in H2228 cells (Fig. 3I and K). qRT-PCR results showed that CCT3 overexpression and knockdown promoted and suppressed the expression of cyclin D1 in LUAD cells, respectively (Fig. 3J and L). These results suggested that CCT3 overexpression contributed to the proliferation and cell cycle progression of LUAD cell.

### CCT3 regulates ferroptosis of LUAD cells

**Fig. 4:**
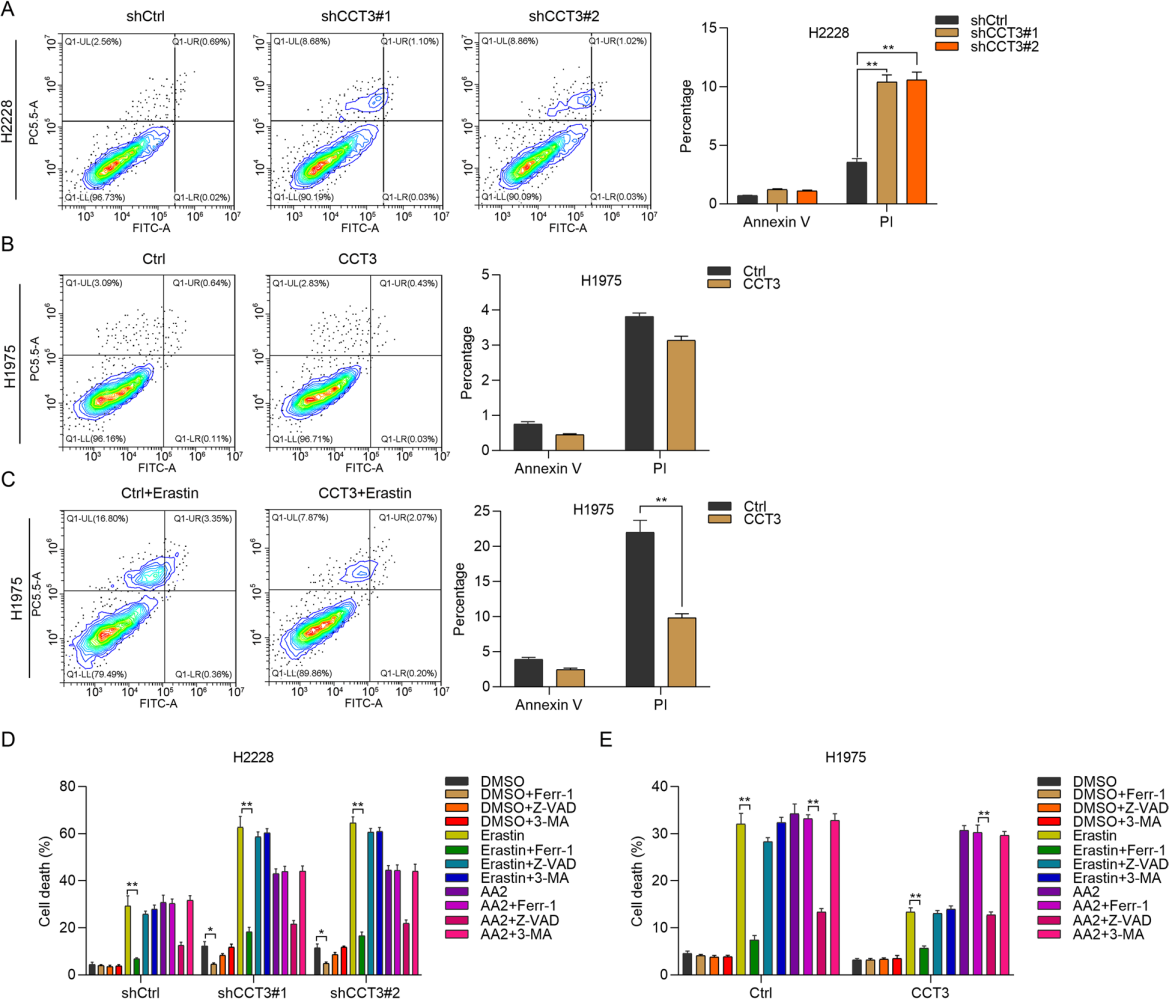
CCT3 inhibits ferroptosis of LUAD cells. **A** shCtrl, shCCT3#1 and shCCT3#2 H2228 cells were subjected to PI/Annexin V-FITC staining and analyzed on flow cytometry. Left, representative images of Annexin V and PI positive cells. Right, quantification results. **p < 0.01. **B** and **C** Ctrl and CCT3 overexpressed H1975 cells treated without erastin (**B**) and with erastin for 8 h (**C**) were subjected to PI/Annexin V-FITC staining and analyzed on flow cytometry. Left, representative images of Annexin V and PI positive cells. Right, quantification results. There results in C showed statistical significance.**p < 0.01. **D** and **E** shCtrl, shCCT3#1 and shCCT3#2 H2228 cells (**D**), Ctrl and CCT3 overexpressed H1975 cells (**E**) were treated with DMSO, ferroptosis inducer erastin (5 uM) and apoptosis activator 2 (5 uM) in combination with or without ferroptosis inhibitor ferrostatin-1 (2 uM), apoptosis inhibitor Z-VAD-FMK (10 ug/ml) or 3-methylademine (2 mM). Then cell viability was examined by trypan blue staining. *p < 0.05. **p < 0.01

Even though CCT3 significantly promoted the growth and proliferation of LUAD cells, CCT3 did not obviously regulate the expression of proliferation markers, including Ki-67 and PCNA. We predicted that CCT3 might modulate the cell death of LUAD cells. To determine whether CCT3 regulates apoptosis, we firstly subjected shCtrl, shCCT3#1 and shCCT3#2 H2228 cells to Annexin V-FITC/PI staining. Stained cells were analyzed on flow cytometry. We found that CCT3 knockdown had no effect on cell apoptosis, but increased the percentage of PI positive cells in H2228 cells (Fig. 4A). However, CCT3 overexpression had minimal effect on the percentage of Annexin V or PI positive cells (Fig. 4B). This could be explained by that Ctrl LUAD cells had low level of cell death. To validate whether CCT3 regulates ferroptosis, we treated H1975 cells transfected with Ctrl or CCT3 lentivirus with ferroptosis inducer erastin and the cells were subjected to Annexin V-FITC/PI staining. The results showed that CCT3 overexpression reduced the percentage of PI positive cells in H1975 cells treated with erastin (Fig. 4C). To further validate CCT3 regulated the cell death depending on ferroptosis but not on other types of cell death, we treated shCtrl, shCCT3#1 and shCCT3#2 H2228 cells with DMSO, ferroptosis inducer erastin (5 uM) and apoptosis activator 2 (AA2, 5 uM) in combination with or without ferroptosis inhibitor ferrostatin-1 (Ferr-1, 2 uM), apoptosis inhibitor Z-VAD-FMK (Z-VAD, 10 ug/ml) or autophagy inhibitor 3-methylademine (3-MA, 2 mM). Cell viability was measured by staining the cells with trypan blue. When H2228 cells were treated with DMSO combination with or without different cell death inhibitors, only ferr-1 had significantly promoting function on the viability of H2228 cells with CCT3 knockdown. CCT3 knockdown significantly promoted the cytotoxicity of erastin but not AA2 in H2228 cells (Fig. 4D, shCtrl + AA2 vs shCCT3#1 + AA2 and shCCT3#2 + AA2, *p* > 0.05). Ferr-1 could reverse the cytotoxicity of erastin on shCtrl or shCCT3 H2228 cells, whereas apoptosis inhibitors Z-VAD or autophagy inhibitors had no such effect (Fig. 4D). On the contrary, Ctrl and CCT3 overexpressed H1975 cells were treated with different cell death inducers or inhibitors similar with H2228 cells. We found that erastin treatment induced high cell death in H1975 cells, which could be reversed by CCT3 overexpression. However, CCT3 overexpression had no effect on the cytotoxicity of AA2 (Fig. 4E, Ctrl + AA2 vs CCT3 + AA2, *p* > 0.05). Collectively, CCT3 has specific role on the ferroptosis of LUAD cells.

### CCT3 transcriptionally activates slc7a11 to suppress ferroptosis

**Fig. 5:**
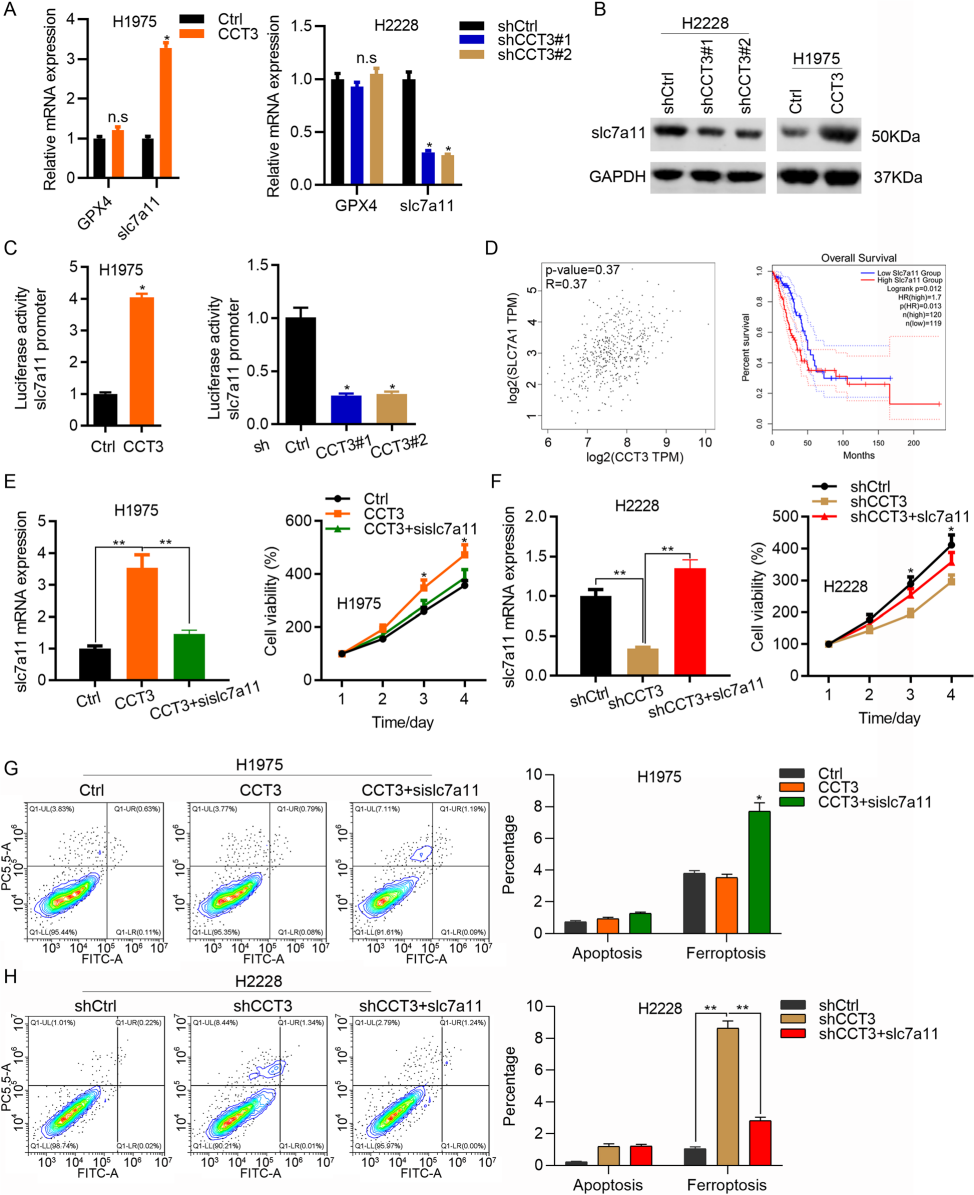
CCT3 upregulation of slc7a11 inhibits ferroptosis. **A** qRT-PCR analysis of GPX4 and slc7a11 in Ctrl and CCT3 overexpressed H1975 cells, and in shCtrl, shCCT3#1 and shCCT3#2 H2228 cells. GAPDH is the internal control. *p < 0.05. **B** Western blot analysis of slc7a11 in cells as shown in A. GAPDH is the internal control. *p < 0.05. **p < 0.01. The blots were cut prior to hybridisation with antibodies during blotting. **C** Dual luciferase activity was checked in H2228 cells transfected with shRNAs (shCtrl, shCCT3#1 and shCCT3#2) or in H1975 cells transfected with pCDNA3.1 plasmids (pCDNA3.1-Ctrl or pCDNA3.1-CCT3), pGL3.Basic-slc7a11 promoter and TK. **D** Sperman correlation between CCT3 and slc7a11 in LUAD patients was analyzed from TCGA. Overall survival of LUAD patients (slc7a11 high and low expression groups) was analyzed from TCGA. **E** qRT-PCR analysis of slc7a11 and CCK8 analysis of cell proliferation were performed in Ctrl, CCT3 and CCT3 + sislc7a11 H1975 cells. *p < 0.05. **p < 0.01. **F** qRT-PCR analysis of slc7a11 and CCK8 analysis of cell proliferation were performed in shCtrl, shCCT3 and shCCT3 + slc7a11 H2228 cells. *p < 0.05. **p < 0.01. **G** and **H** Apoptosis and ferroptosis were detected by using Annexin V-FITC/PI staining in Ctrl, CCT3 and CCT3 + sislc7a11 H1975 cells (**G**), and in shCtrl, shCCT3 and shCCT3 + slc7a11 H2228 cells. Left, representative images of apoptosis and necroptosis. Right, quantification results. *p < 0.05. **p < 0.01

GPX4 and slc7a11 are essential ferroptosis regulators. We then explored whether CCT3 regulated GPX4 and slc7a11. Based on qRT-PCR results, CCT3 overexpression or knockdown had no effect on the expression of GPX4 (Fig. 5A). By contrast, CCT3 overexpression promoted the mRNA expression of slc7a11, while its knockdown suppressed the mRNA expression of slc7a11 (Fig. 5A). Consistent results were observed on the protein expression of slc7a11 (Fig. 5B). These results suggested that CCT3 positively regulated the expression of slc7a11 at transcriptional manner. To address this question, we performed dual luciferase activity assay when interfering or overexpressing CCT3. We showed that CCT3 knockdown reduced, while the overexpression enhanced the luciferase activity of slc7a11 promoter (Fig. 5C). Importantly, CCT3 was positively correlated slc7a11 in LUAD patients. Overexpression of slc7a11 predicted poor prognosis of LUAD patients (Fig. 5D). To validate the role of CCT3/slc7a11 axis in LUAD growth and ferroptosis, slc7a11 was silenced by siRNAs transfection in CCT3 overexpressed H1975 cells and was ectopically expressed in CCT3 silenced H2228 cells. As expected, slc7a11 downregulation inhibited the growth of CCT3 overexpressed H1975 cells (Fig. 5E). By contrast, slc7a11 overexpression promoted the growth of CCT3 silenced H2228 cells (Fig. 5F). Furthermore, slc7a11 knockdown resulted in increased ferroptosis in CCT3 overexpressed H1975 cells (Fig. 5G). Slc7a11 overexpression reversed the ferroptosis induced by CCT3 knockdown in H2228 cells (Fig. 5H). Therefore, CCT3 upregulation of slc7a11 suppresses the ferroptosis and promotes the proliferation of LUAD cells.

### CCT3 activates AKT signaling pathway in LUAD cells.

**Fig.6:**
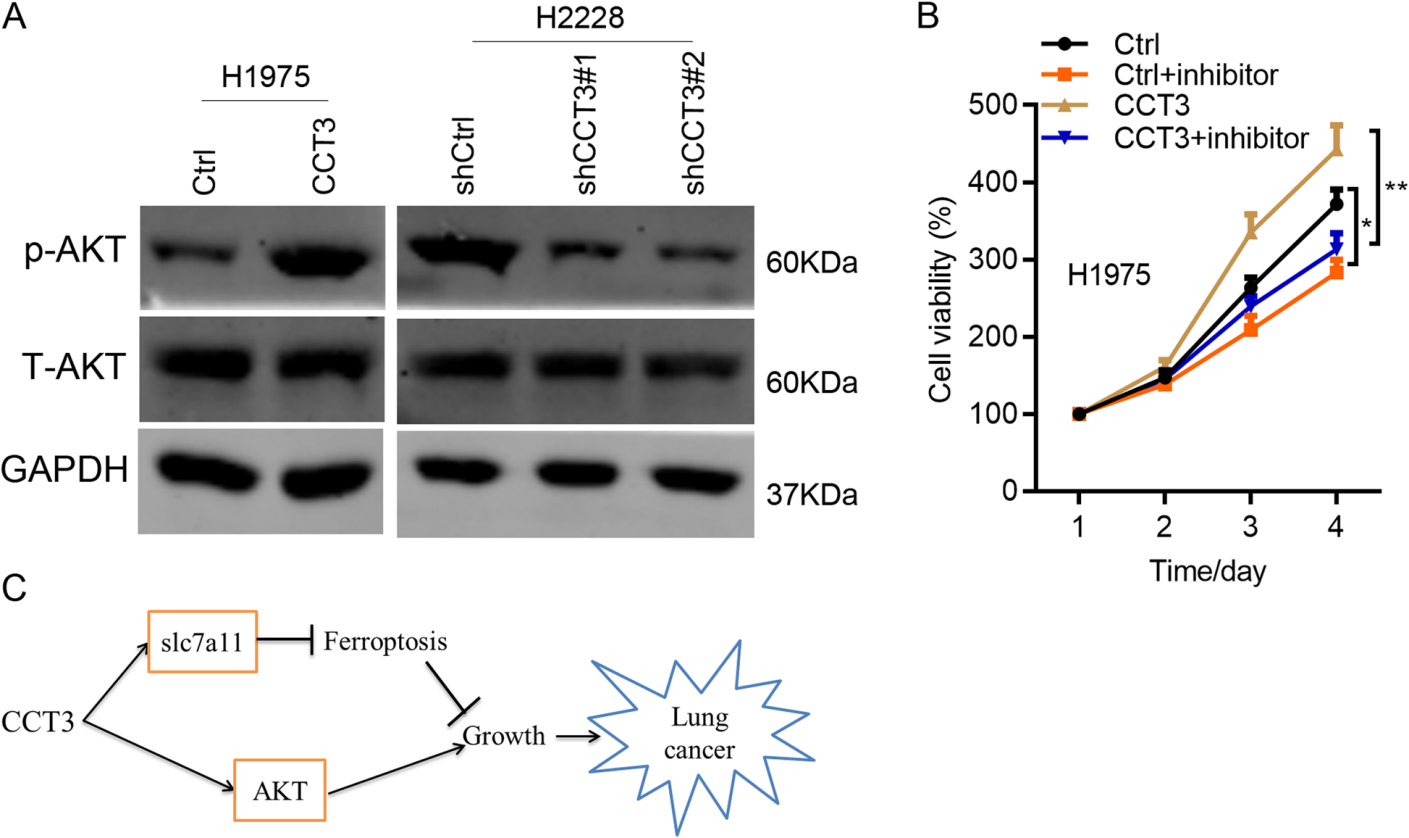
CCT3 stimulates AKT activity. **A** Ctrl and CCT3 overexpressed H1975 cells, shCtrl, shCCT3#1 and shCCT3#2 H2228 cells were subjected Western blot analysis with indicated antibodies. The blots were cut prior to hybridisation with antibodies during blotting. **B** Cell viability was the determined by CCK8 assay in Ctrl, CCT3 overexpressed, Ctrl and CCT3 overexpressed H1975 cells that were treated with MK2206 (2 uM) for indicated time. *p < 0.05. **p < 0.01. **C** Schematic diagram of how CCT3 contributes to LUAD cell growth.

Above results suggested that CCT3 knockdown significantly suppressed cell proliferation through promoting the ferroptosis of LUAD cells. However, CCT3 overexpression had minimal effect on the ferroptosis of H1975 cells when treated without erastin (Fig. 4B and E). Therefore, there might be another downstream substrate or signaling pathway which contributed to the oncogenic role of CCT3 in LUAD. AKT/mTOR signaling pathway is hyper-active in various cancers, including LUAD. We subjected CCT3 knockdown, overexpression and control cells to Western blot analysis of AKT activity. The results showed that knockdown of CCT3 suppressed the phosphorylation, while had no effect on the expression of AKT in H2228 cells (Fig. 6A). By contrast, CCT3 overexpression activated AKT in H1975 cells (Fig. 6A). Furthermore, we used MK2206, a specific AKT inhibitor, to treat H1975 cells without or with CCT3 overexpression. We found that MK2206 more obviously inhibited the proliferation of CCT3 overexpressed H1975 cells (Fig. 6B). These results suggest that CCT3 activates AKT in LUAD cells.

Overall, CCT3 promotes LUAD cell growth through inhibition of slc7a11-mediated ferroptosis and activation of AKT (Fig. 6C).

## Discussion

In this present study, we investigated the clinical relevance and function of CCT3 in LUAD. We found that CCT3 was highly expressed in lung cancer tissues. Overexpression of CCT3 predicted poorer overall and disease-free survival of LUAD patients than CCT3 low expression. Functional experiments showed that CCT3 expression was critical for the proliferation and growth of LUAD cells. Slc7a11-mediated ferroptosis was suppressed by CCT3. Furthermore, CCT3 activated AKT. AKT specific inhibitor MK2206 suppressed the proliferation of LUAD cells. Therefore, we reveal that CCT3 promotes LUAD cell proliferation and growth through negative regulating ferroptosis and positively regulating AKT signaling cascade.

As a subunit of TRiC family, CCT3 plays a pivotal role in cancer progression. CCT3 is overexpressed in the tissues and serum of HCC patients. Upregulation of CCT3 promotes the development of HCC by regulating cell cycle and DNA replication pathways [12, 13]. In gastric cancer, CCT3 is highly expressed in the cancer tissues compared with normal tissues. CCT3 augments the viability of gastric cancer cells by modulating cell cycle proteins, including mitogen-activated protein kinase 7, cyclin D3, and cyclin-dependent kinases [14]. In addition, CCT3 is also overexpressed in papillary thyroid carcinoma (PTC) specimens. Knockdown of CCT3 significantly suppresses the proliferation and cell cycle progression of PTC cells [17]. Another study demonstrated that knockdown of CCT3 blunted the proliferation and migration of breast cancer cells by inhibiting NF-κB signaling. Overexpression of NF-κB reversed the inhibitory effect of CCT3 silencing on breast cancer cell proliferation and migration [18]. Recently, the role of CCT3 in lung cancer has been addressed. For example, CCT3 overexpression promoted lung cancer cell growth and migration through regulating YAP1 [15]. In another study, the results showed that CCT3 knockdown induced cell apoptosis in lung cancer cells and promotes the sensitivity of cisplatin on A549 cells [16]. In this study, we showed that CCT3 was upregulated in lung cancer tissues based on TCGA database and our qRT-PCR and immunoblotting analysis. CCT3 was also upregulated in lung cancer cells compared with lung epithelial cells. We also showed that CCT3 expression was inversely correlated with the overall and disease-free survival of LUAD patients. These results indicate that CCT3 is a potential biomarker for LUAD. Furthermore, we demonstrated that CCT3 knockdown suppressed, while CCT3 ectopic expression promoted the proliferation and colony formation of LUAD cells. Unlike other studies which demonstrated that CCT3 regulated the apoptosis of lung cancer cells, such as A549 and H520 cells, we observed that CCT3 had no effect on the apoptosis of H2228 and H1975 cells. Instead, ferroptosis of the H2228 and H1975 cells treated with erastin was increased and decreased by CCT3 knockdown and overexpression, respectively. There were some reasons to possibly explain what caused the difference between others’ and our studies: 1, the regulation pattern among different cancer subtypes or cell types was different. 2, the molecular characteristics between A549 and H520 cells versus H2228 and H1975 cells were different. Collectively, these results highlight the oncogenic role of CCT3 functions in LUAD.

In recent years, ferroptosis was reported as an important subtype of cell death during cancer development [19]. Inhibition of ferroptosis promotes carcinogenesis in different cancers, including lung cancer, breast cancer and melanoma [20–22]. As an essential ferroptosis repressor, slc7a11 was overexpressed in some cancer tissues and contributed to cancer growth. In addition, slc7a11 was regulated by various oncogenes or tumor suppressors. For example, loss-of-function of P53 inhibits ferroptosis and promotes cancer growth through upregulation of slc7a11 [23]. Slc7a11 can also be inhibited by radiotherapy or immunotherapy [24]. In this study, we identified CCT3 as a novel regulator of slc7a11. CCT3 upregulation of slc7a11 suppressed ferroptosis but not apoptosis. These results provide a newly evidence that CCT3 serves as ferroptosis suppressor through regulating the expression of slc7a11.

AKT serine/threonine kinase is an important regulator of cellular metabolism, including fatty acid, glucose and amino acid [25]. It acts as a pivotal oncogene by promoting the survival and migration of different cancer cells [26]. Hyper-activation of AKT is frequently observed in NSCLC patients due to the gain-of-function mutation of PI3KCA and AKT [27]. Identifying novel regulators of AKT signaling may help us formulate AKT inhibitor treatment for the appropriate patients. Here, we showed that CCT3 potentiated AKT activity in LUAD cells. Importantly, AKT inhibitor, MK2206, significantly suppressed the growth in CCT3 highly expressed lung cancer cells. Our study not only indicates that CCT3 activates AKT to promotes LUAD, but also shows that CCT3 expression level is important for efficacy of AKT inhibitors in lung cancer cells.

There were some limitations in this study. 1. We did not deeply explore the regulation of CCT3 on the ferroptosis of LUAD cells. 2. We did not investigate the clinical significance of CCT3/slc7a11/ferroptosis in large-scale samples. 3. We did not illustrate the role of CCT3/slc7a11/ferroptosis axis in a preclinical animal model. In the future, we will perform intensive studies to address these questions.

## Conclusion

Overall, we present the evidences that CCT3 functions as an oncogene in LUAD. CCT3 is highly expressed in LUAD tissues and confers poor prognosis of LUAD patients. Overexpression of CCT3 promotes the proliferation and growth of lung cancer cells. In addition, CCT3 inhibits ferroptosis through positive regulation of slc7a11. Importantly, CCT3 activates AKT and AKT inhibitor MK2206 could reverse the oncogenic role of CCT3. Therefore, targeting AKT may be effective for lung cancer patients with highly expressed CCT3. Combination of AKT inhibitor and ferroptosis inducer could be a promising therapeutic strategy for the treatment of LUAD.

## Methods

### Cell lines and reagents

Lung epithelial cells Beas-2B, lung large cell carcinoma cells H1299, and lung adenocarcinoma cells H1975 and H2228 were obtained from American Type Culture Collection (Manassas, VA, USA). The cells were maintained in Dulbecco's Modified Eagle Medium or RPMI 1640 Medium (Gibco, California, USA), containing 10% fetal bovine serum (Gibco) and 1% antibiotics (Corning, New York, USA). Cell culture was conducted in a 37 °C incubator with 5% CO_2_. AKT inhibitor MK2206 was purchased from Selleckchem (TX, USA). Ferrostatin-1 was obtained from Xcess Biosciences. Other drugs were from Sigma-Aldrich. Antibodies against p-AKT and T-AKT were obtained from Cell Signaling Technology (Danvers, USA). Slc7a11 primary antibody was from Abcam (Cambridge, United Kingdom). GAPDH primary antibody and all the secondary antibodies were from Proteintech (Chicago, USA).

### Human lung cancer tissues and The Cancer Genome Atlas

Human lung cancer tissues and adjacent tissues were collected from the patients before any therapeutic intervention at Kunming Fourth People's Hospital, Seventh Affiliated Hospital of Dali University. Written informed consent was obtained from the patients. The experiments were approved by Ethics Committee of Kunming Fourth People's Hospital, Senventh Affiliated Hospital of Dali University (ky201620) and performed according to the World Medical Association Declaration of Helsinki. The mRNA and protein expression of CCT3 was analyzed in these tissues.

Transcript abundance of CCT3, overall survival, and disease-free survival of LUAD and LUSC were analyzed from http://gepia.cancer-pku.cn/. For overall and disease-free survival analysis, LUAD patients were divided into CCT3 high and low group. The patient number was 120 and 239 in each group when the data analysis was performed using Quartile and Median, respectively. LUSC were divided into CCT3 high and low group. The patient number was 121 and 241 in each group when the data analysis was performed using Quartile and Median, respectively.

### CCT3 knockdown and overexpression

Lentivirus system was applied to knock down and overexpress CCT3. For CCT3 knockdown, short hairpin RNAs (shRNA) targeting CCT3 was cloned into pLKO1.1-puro vector. The shRNA targeting sequence of CCT3 was as follow: shCCT3-1: 5’-GCCAAGTCCATGATCGAAATT-3’ and shCCT3-2: 5’-GCTACTGCGAATTGATGACAT-3’. For CCT3 or slc7a11 overexpression, the coding sequence of CCT3 or slc7a11 was cloned into pLV105 plasmid. To package lentivirus, psPAX2 and pLV-VSVG vectors were co-transfected with the lentivirus vectors into 293FT cells. The virus were harvested 48 h later and used to infect H2228 and H1975 cells. Puromycin was used to construct stable cell lines with CCT3 knockdown and overexpression.

### Quantitative real-time PCR (qRT-PCR)

Human lung cancer tissues or indicated cells were subjected to total RNA extraction using TRIzol reagent (Invitrogen, Carlsbad, CA, USA), according to the manufacturer's protocols. Then, 1 ug of the RNA was reversely transcribed into cDNA using RT-for-PCR kit (Clontech, Takara, Japan). Quantification of mRNA level was performed using SYBR Green II (Takara, Japan). The primer sequence was as follow: CCT3 forward, 5’-TCAGTCGGTGGTCATCTTTGG-3’ and reverse, 5’-CCTCCAGGTATCTTTTCCACTCT-3’; Ki-67 forward, 5’-AGAAGAAGTGGTGCTTCGGAA-3’ and reverse, 5’-AGTTTGCGTGGCCTGTACTAA-3’; PCNA forward, 5’-CCTGCTGGGATATTAGCTCCA-3’ and reverse, 5’-CAGCGGTAGGTGTCGAAGC-3’; Cyclin D1 forward, 5’-CAATGACCCCGCACGATTTC-3’ and reverse, 5’-CATGGAGGGCGGATTGGAA-3’; GPX4 forward, 5’-GAGGCAAGACCGAAGTAAACTAC-3’ and reverse, 5’-CCGAACTGGTTACACGGGAA-3’; GAPDH forward, 5’-TGTGGGCATCAATGGATTTGG-3’ and reverse, 5’-ACACCATGTATTCCGGGTCAAT-3’. The 2^−ΔΔCt^ method was applied to determine mRNA expression and GAPDH acted as the internal control.

### Immunoblotting

Total protein was extracted from human tissues and cells using RIPA lysis buffer (Beyotime Biotechnology, Shanghai, China), which was supplemented with protease and phosphatase inhibitor cocktail (Roche, Basel, Switzerland). 30–50 ug of the protein was separated on SDS- polyacrylamide gel electrophoresis (SDS-PAGE) and transferred onto PVDF membranes. After blocking with 5% non-fat milk for 1 h, the membranes were incubated with primary antibodies at 4 °C overnight and with secondary antibodies at room temperature for 3 h. Protein abundance was detected by Chemiluminescent ECL reagent (Beyotime Biotechnology). Antibodies against CCT3 (60,264–1-Ig) and GAPDH (60,004–1-Ig) were obtained from Proteintech. Slc7a11 antibody (PA1-16,893) was from Invitrogen. p-AKT (#4060) and AKT (#4691) primary antibodies were purchased from Cell Signaling Technology. Mouse (SA00001-1) and rabbit (SA00001-2) secondary antibodies were from Proteintech.

### Cell proliferation and ferroptosis detection

Cell counting kit-8 (CCK8) kit (YEASEN, Shanghai, China) was used to detect cell proliferation. Indicated lung cancer cells were seeded into 96-well plates in triplicate at the density of 2000 cells per well. 6, 30, 54 and 78 h later, the OD value at 450 nm was detected. Cell viability at 6 h after seeding was recognized as day 1. Cell proliferation was normalized to the OD450 value of day 1.

To determine ferroptosis, we treated the cells with DMSO, ferroptosis inducer erastin (5 uM) and apoptosis activator 2 (5 uM) in combination with or without ferroptosis inhibitor ferrostatin-1 (2 uM), apoptosis inhibitor Z-VAD-FMK (10 ug/ml) or 3-methylademine (2 mM). Then cell viability was examined by trypan blue staining.

### Colony formation assay

For CCT3 knockdown, a total of 2000 shCtrl, shCCT3#1 and shCCT3#2 H2228 cells were seeded into 6-well plates. For CCT3 overexpression, a total of 800 Ctrl and CCT3 overexpressed H1975 cells were seeded. 10 days later, colonies were washed with PBS and stained with crystal violet solution. Images of colonies were photographed under the camera.

### Apoptosis and cell cycle detection

Apoptosis of LUAD cells was measured using PI/Annexin V-FITC kit (Invitrogen). In brief, control, CCT3 silenced H2228 cells and CCT3 overexpressed H1975 cells in 6-well plates in triplicate were trypsinized and washed by PBS. After stained with PI and Annexin V-FITC, apoptosis was detected on the flow cytometer (BECKMAN COULTER, Minnesota, USA), according to the manufacturer's protocols.

Cell cycle of LUAD cells was measured using PI kit (YEASEN, Shanghai, China) and was detected on the flow cytometer (BECKMAN COULTER), according to the manufacturer's protocols.

### Dual luciferase reporter assay

The promoter sequence was cloned into pGL3.basic vector. Coding sequence of CCT3 was inserted into pCDNA3.1. After transfecting pCDNA3.1 and shRNAs, accompanied with pGL3.basic and TK vectors to H1975 and H2228 cells, dual luciferase activity was assessed by using Dual-Luciferase Reporter Assay Kit (Promega, USA), according to manufacturers’ instructions. The luciferase activity was adjusted to Renilla luciferase expression vector, pCMV-RL-TK.

### Statistical analysis

All the data were presented as mean ± standard error of the mean (SEM) and were analyzed using GraphPad Prism. Student’s t test was used to compare the difference between two groups. One-way ANOVA was applied when more than two groups. Statistical difference was considered significantly when P was less than 0.05.

## Supplementary Information

**Additional file 1: Supplementary Figure1. **CCT3 expression is not associated with the survival of LUSC patients. (A)Overall survival of LUSC patients with high expression and low expression ofCCT3. *n*=121 in each group. *p*=0.29. (B) Disease free survival ofLUSC patients with high expression and low expression of CCT3. *n*=121 in each group. *p*=0.68. The data analysis was performedusing Quartile. (C) Overall survival of LUSC patients with high expression andlow expression of CCT3. *n*=241 in eachgroup. *p*=0.97. (D) Disease freesurvival of LUSC patients with high expression and low expression of CCT3. *n*=241 in each group. *p*=0.61. The data analysis was performedusing Median. Original WB bands.

## Data Availability

All of the data generated during this study were included in this article.
