# Supplementary material for: Upregulation of CCT3 predicts poor prognosis and promotes cell proliferation via inhibition of ferroptosis and activation of AKT signaling in lung adenocarcinoma
Source: BMC Mol Cell Biol. 2022 Jun 30;23:25. doi: 10.1186/s12860-022-00424-7 (PMC9245217; doi:10.1186/s12860-022-00424-7)
Supplement: Supplementary file 1 — Additional file 1: Supplementary Figure1. CCT3 expression is not associated with the survival of LUSC patients. (A)Overall survival of LUSC patients with high expression and low expression ofCCT3. n=121 in each group. p=0.29. (B) Disease free survival ofLUSC patients with high expression and low expression of CCT3. n=121 in each group. p=0.68. The data analysis was performedusing Quartile. (C) Overall survival of LUSC patients with high expression andlow expression of CCT3. n=241 in eachgroup. p=0.97. (D) Disease freesurvival of LUSC patients with high expression and low expression of CCT3. n=241 in each group. p=0.61. The data analysis was performedusing Median. Original WB bands. [file 12860_2022_424_MOESM1_ESM.docx]

**Supplementary Figure 1. CCT3 expression is not associated with the survival of LUSC patients.**

**
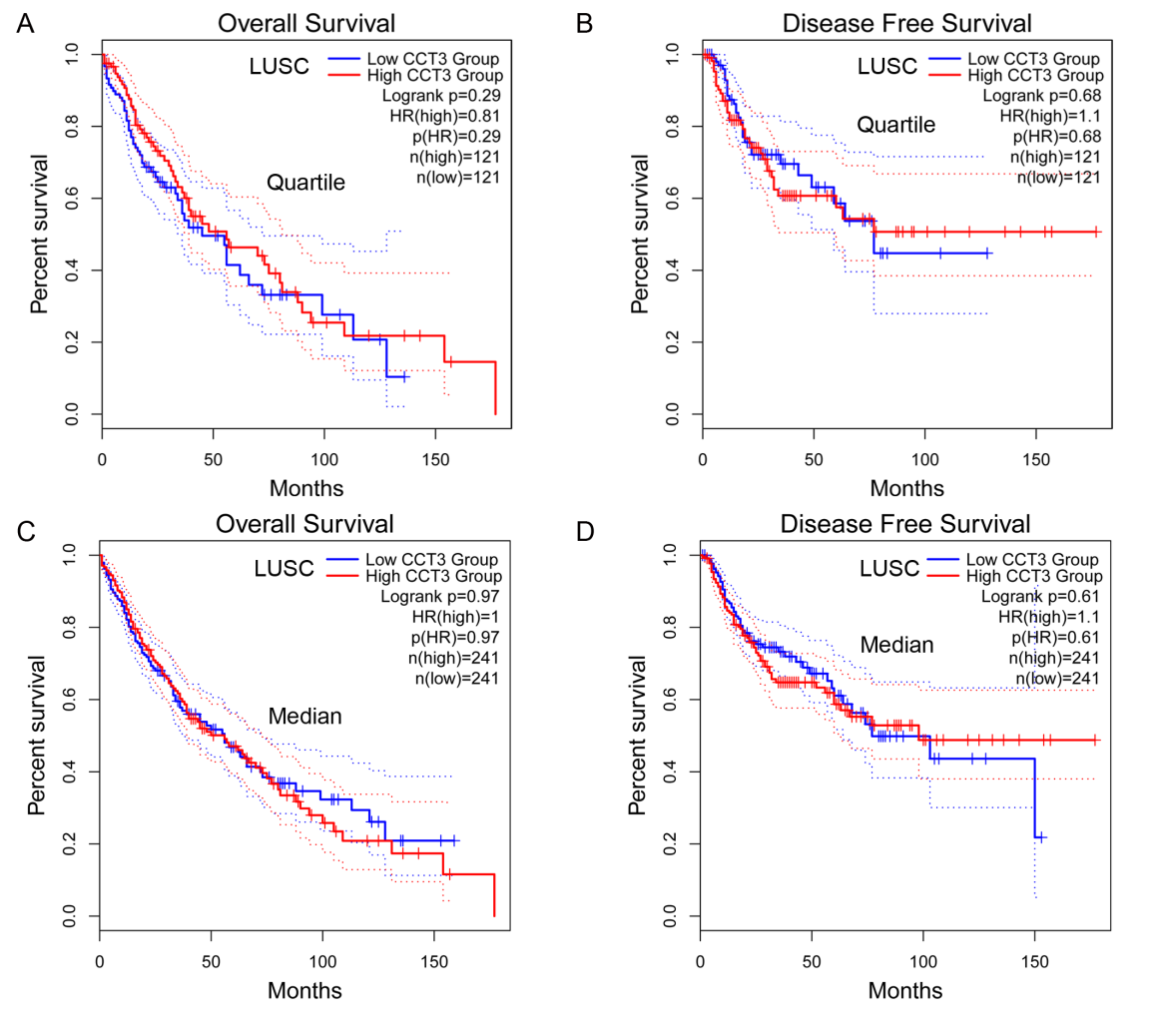
**

(A) Overall survival of LUSC patients with high expression and low expression of CCT3. n=121 in each group. p=0.29. (B) Disease free survival of LUSC patients with high expression and low expression of CCT3. n=121 in each group. p=0.68. The data analysis was performed using Quartile. (C) Overall survival of LUSC patients with high expression and low expression of CCT3. n=241 in each group. p=0.97. (D) Disease free survival of LUSC patients with high expression and low expression of CCT3. n=241 in each group. p=0.61. The data analysis was performed using Median.

**Original WB bands**

**
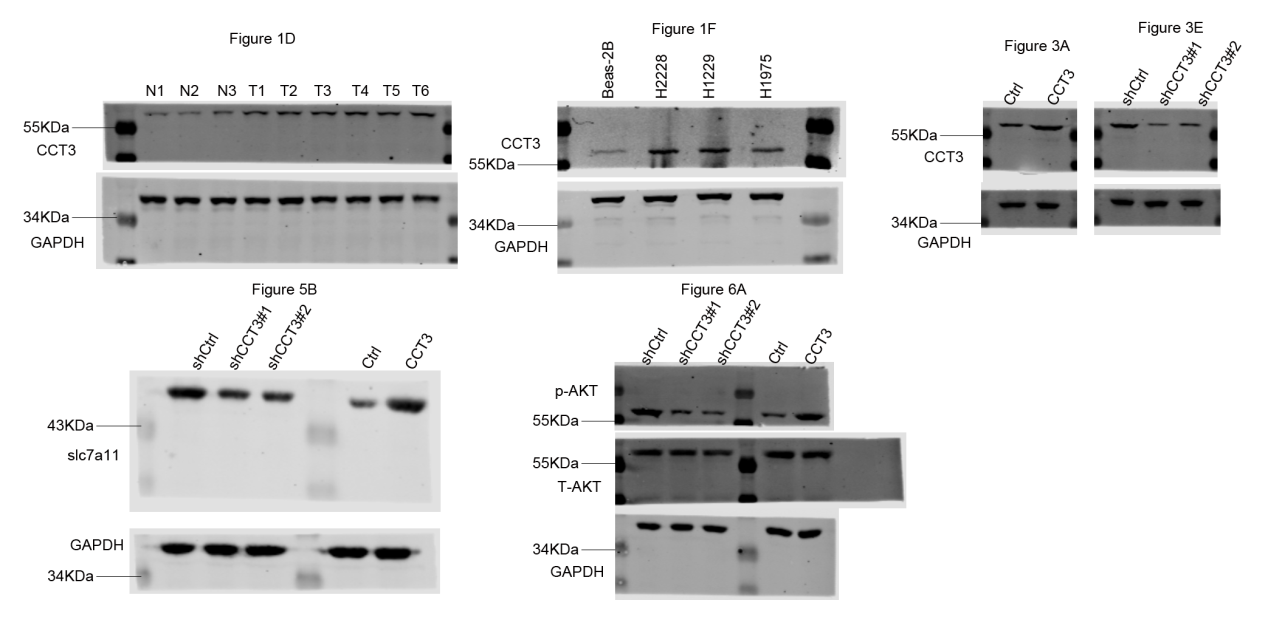
**
